# Death by community-based methicillin-resistant *Staphylococcus aureus*: case report

**DOI:** 10.5935/2965-2774.20230078-en

**Published:** 2023

**Authors:** Júlia Lima Vieira, Ruy Pezzi de Alencastro, Francisco Bruno, Taís Sica da Rocha, Jefferson Pedro Piva

**Affiliations:** 1 Pediatric Intensive Care Unit, Hospital Moinhos de Vento - Porto Alegre (RS), Brazil; 2 Pediatric Intensive Care Unit, Hospital de Clínicas de Porto Alegre, Universidade Federal do Rio Grande do Sul - Rio Grande do Sul (RS), Brazil

## INTRODUCTION

Community methicillin-resistant *Staphylococcus aureus* (CA-MRSA) has by definition a minimum inhibitory concentration for oxacillin ≥ 4mcg/mL, giving it intrinsic resistance to all beta-lactams, including cephalosporins, which is associated with the presence of the *mecA* gene. It also has bacteriological and epidemiological characteristics distinct from hospital-acquired MRSA, including its resistance profile to other antimicrobials, its genotypic lineage, its genetic element that encodes methicillin resistance, and its toxin production profile.^(1)^ There are few data on the prevalence of CA-MRSA in Brazil. Carvalho et al. identified a high rate of CA-MRSA colonization (7.4%) in healthy children attending day care centers in northeastern Brazil.^(2)^ Gelatti et al. evaluated 104 samples from patients hospitalized with cutaneous infections in the community in southern Brazil, 58 of which were *S. aureus* isolates; of these, 8.6% were CA-MRSA.^(3)^ CA-MRSA has been increasing worldwide in prevalence, causing concern due to its ability to cause fatal infections.^(4)^ A study conducted in Cameroon showed a 20-30% increase in its prevalence in 2003, and the increase had reached 80% in 2019.^(5)^ A meta-analysis of population prevalence studies in cities and regions of the United States revealed a dramatic increase in CA-MRSA infections in the last two decades, with CA-MRSA endemic strains at unprecedented levels in many regions of the United States in a heterogeneous pattern among regions, which seems to have occurred earlier in children than adults.^(6)^

The CA-MRSA is transmitted through contact with a colonized individual or a contaminated surface, especially in healthy children and adolescents. The CA-MRSA clones may be more efficient than other strains in colonizing the human body and surviving on surfaces. Risk factors include situations of frequent physical contact, rupture of skin integrity, sharing of items, poor housing and hygiene conditions, crowding, sexual habit (sex between men), and exposure to various antibiotics. The risk of infection significantly increases with colonization.^(4,7,8)^

Among the invasive infections caused by CA-MRSA, necrotizing pneumonia is rare but has high morbidity and mortality. Records in the United States and Europe report mortality above 50%, affecting healthy adolescents and young adults.^(1)^ In reporting this case of a healthy adolescent with fulminant progression of CA-MRSA treated at a tertiary hospital in Porto Alegre, Rio Grande do Sul, Brazil, we intended to alert to the occurrence of this rare event in our country, as well as to discuss the various anatomopathological findings.

## CASE REPORT

A previously healthy male patient (13 years old, weighing 40kg) came down with a headache, odynophagia, and dry cough. On the second day, the patient developed fever, progressing to dyspnea and chest pain. The patient was seen in the emergency room, where tonsillitis was diagnosed, and as a symptomatic patient was discharged with a prescription of prednisolone and amoxicillin. On the fourth day, the patient presented worsening odynophagia accompanied by mild dyspnea and one episode of vomiting. In the late morning, his school teacher activated the Mobile Emergency Care Service (SAMU - *Serviço de Atendimento Móvel de Urgência*) due to pallor, dyspnea, and cervical bulging. On arrival at the hospital, the patient presented tachypnea, intense respiratory effort, poor peripheral perfusion, and weak pulses, in addition to cervical and thoracic subcutaneous emphysema. The patient evolved to ventilatory failure and needed orotracheal intubation. After intubation, there was bleeding from the tracheal tube. A chest X-ray showed bilateral opacities and extensive subcutaneous emphysema. Bilateral chest drainage was performed due to suspicion of hemopneumothorax, with bloody discharge. Noradrenaline was initiated, and the patient was referred to the pediatric intensive care unit (ICU) of a tertiary hospital.

**Figure 1:**
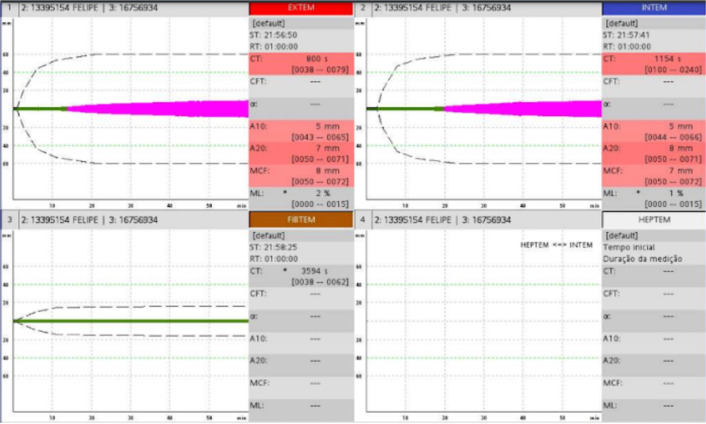
Thromboelastogram.

**Figure 2:**
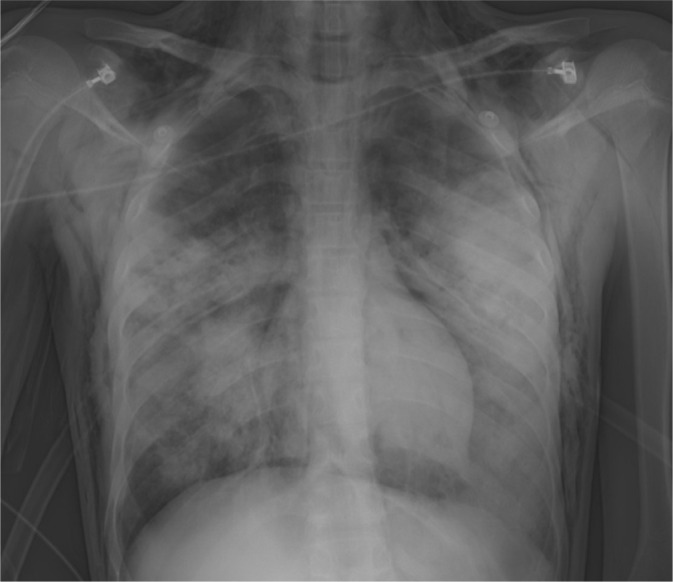
Chest X-ray.

Upon arrival at the pediatric ICU, cardiorespiratory arrest was observed in asystole, and cardiopulmonary resuscitation was initiated immediately. The tracheal tube was in the proper position, but there was profuse bleeding on aspiration and much rubbery gastric residue in the oral cavity. He returned to sinus rhythm 6 minutes later, and epinephrine was started as a continuous infusion. Immediate transfusion of plasma, red blood cells, and platelets was requested. A bolus of tranexamic acid was initiated, followed by continuous infusion, in addition to antibiotic therapy with cefepime and clindamycin, due to the severity and suspicion of aspiration due to a history of vomiting. Postarrest physical examination showed poor peripheral perfusion, symmetrical myotic pupils, and extensive subcutaneous emphysema in the neck, chest, and upper limbs. Laboratory tests were performed (Table 1), including thromboelastogram (Figure 1) and chest X-ray (Figure 2). The X-ray showed extensive pulmonary consolidative opacities, pneumomediastinum and extensive subcutaneous emphysema. One hour after admission, he developed anisocoria and was managed with mannitol and hyperventilation. He developed a new cardiorespiratory arrest, with pulseless electrical activity, which reversed after a cycle and after a dose of adrenaline. He received a second dose of mannitol, and continuous hypertonic solution was initiated, reversing his anisocoria.

**Table 1:** Test results

Exams (reference values)	
Hematocrit (36 - 58%)	34.9%
Hemoglobin (11.6 - 15.6g/dL)	11.2g/dL
Leukocytes (3.6 - 11.0 × 103/µL)	950/µL (Poles 3%; segmented 20%; Lymphocytes 66%)
Platelets (150 - 400 × 103/µL)	25000/µL
RNI (< 1.2)	3.76
Prothrombin time (> 70%)	21%
Activated partial thromboplastin time (29 - 38 seconds)	165 seconds
Fibrinogen (200 - 400mg/dL)	113mg/dL
Blood gas analysis	
pH (7.35 - 7 .45)	pH 6.81
pCO_2_ (38 - 50mmHg)	pCO_2_ 117mmHg
HCO_3_ (22 - 26mmol/L)	HCO_3_ 18mmol/L
sVO_2_ (95 - 100%)	sVO_2_ 34%
Lactate (0.5 - 1.6 mmol/L)	14 mmol/L
Troponin (< 34.2pg/mL)	135pg/mL
BNP (< 100pg/mL)	120pg/mL
Aspartate aminotransferase (13 - 35U/L)	136U/L
Alanine aminotransferase (8 - 24U/L)	46U/L
C-reactive protein (< 5mg/L)	89mg/L
Creatinine (0.57 - 0.8mg/dL)	2.23mg/dL
Urea (15 - 36mg/dL)	73mg/dL
Sodium (136 - 146mEq/L)	140mEq/L
Potassium (3.4 - 3.5mEq/L)	3.5mEq/L
Magnesium (1.7 - 2.2mg/dL)	3mg/dL
Phosphorus (2.3 - 4.7mg/dL)	10.3mg/dL
Ionic Calcium (4.6 - 5.3mg/dL)	4.9mg/dL
Chlorine (98 - 107mEq/L)	106mEq/L

INR - International Normalized Ratio; pCO_2_ - partial pressure of carbon dioxide; HCO_3_ - bicarbonate; sVO_2_ - venous oxygen saturation; BNP - brain natriuretic peptide.

Findings: marked deficiency of intrinsic and extrinsic pathway factors; fibrinogen deficiency; platelet deficiency/dysfunction. Test characteristic of disseminated intravascular coagulation.

Extensive bilateral confluent pulmonary opacities predominantly in the middle and lower fields; pneumothorax; pneumomediastinum; extensive bilateral subcutaneous emphysema.

Vasopressin and dobutamine were associated with persistent hypotension requiring high doses of vasopressor, without hemodynamic stabilization. There was still profuse bleeding in the endotracheal tube, even after transfusion of blood products. His thromboelastogram was compatible with disseminated intravascular coagulation. He needed mechanical ventilation at high parameter settings, which still did not maintain adequate oxygen saturation.

The patient was maintained with pleural drains in continuous aspiration. The otorhinolaryngology department was called in due to suspicion of tonsillar abscess with progression to adjacent soft tissues, but direct laryngoscopy and oroscopy did not show major problems.

The performance of fiberoptic bronchoscopy at the bedside was discussed with pediatric surgeons to identify the cause of bleeding as well as to determine whether he was indicated for installation of an extracorporeal membrane oxygenator. However, the patient deteriorated rapidly in the form of massive bleeding in the endotracheal tube, progressive hypoxemia, and refractory hypotension, without responding to any therapeutic measure. The patient died 5 hours after arrival to the pediatric ICU. Later, in the peripheral blood culture collected on arrival to the pediatric ICU, growth of oxacillin-resistant *S. aureus* sensitive to other antibiotics and of multisensitive *Haemophilus influenzae* was detected.

At necropsy, the following findings stood out: necrotizing pneumonia due to S. aureus associated with diffuse alveolar hemorrhage; lower airway bleeding; massive subcutaneous emphysema, pneumomediastinum, and pleural and pericardial effusions; H. influenzae coinfection; hypocellular bone marrow with depletion of the granulocytic series and hemophagocytosis; mixed shock with septic and hypovolemic components; disseminated intravascular coagulation; and multiorgan ischemic changes associated with end-stage shock, present in the central nervous system, kidneys, liver, gallbladder, pancreas, and small intestine.

The anatomopathological evaluation concluded that the tissue necrosis caused by the bacterial agent led to the loss of integrity of the bronchial tree, with consequent leakage of air into the tissues, triggering massive subcutaneous emphysema and pneumomediastinum. The extensive tissue necrosis also affected vascular walls, triggering diffuse alveolar hemorrhage and lower airway hemorrhage.

## DISCUSSION

When methicillin-resistant *Staphylococcus aureus* was first described in 1961, it was considered a nosocomial pathogen. This perception has changed significantly in the last two decades.^(7,9)^ The first definitive report of CA-MRSA was in 1993, in an Aboriginal population of Australia that had had no contact with major centers and that had a different strain profile from those previously identified. In the same decade, between 1997 and 1999, four children died of sepsis or necrotizing pneumonia caused by CA-MRSA in the Midwest region of the United States. Since then, the epidemiology of CA-MRSA has been changing worldwide, drawing attention due to its rapid emergence, increase in prevalence, and potential to cause serious invasive infections in young and healthy patients.^(7,9,10)^

CA-MRSA strains exhibit virulence factors that neutralize the immune system response and delay the adaptive response, promoting bacterial dissemination in organs and tissues. They produce high concentrations of cytolytic peptides that recruit, activate, and lyse neutrophils. Among them, Panton-Valentine leukocidin (PVL) is an exotoxin frequently found in CA-MRSA strains, encoded by the lukS-PV and lukF-PV genes. It has toxic and immunomodulatory properties and is associated with cutaneous soft tissue infections and severe necrotizing pneumonia. It mainly targets neutrophils, monocytes and macrophages, connecting to receptors on the membrane of these cells, inducing pore formation and leading to cell destruction. It also induces the release of pro-inflammatory cytokines, being an important virulence factor associated with necrotizing pneumonia.^(11,12)^

The prevalence of PVL-producing *S. aureus* is quite variable and is associated with certain strains and lineages, especially in CA-MRSA. Testing is quite restricted, being rarely performed outside reference centers, being also underrepresented and inaccurate.^(9)^ In a Brazilian study that evaluated the national registries related to CA-MRSA, the genes associated with PVL were identified in 100% of the identified strains.^(8)^

The clinical spectrum of CA-MRSA infections includes soft tissue infections and invasive infections, which can be spontaneous or result from skin lesions. Invasive lesions include septic arthritis, bacteremia without focus, necrotizing pneumonia, meningitis, necrotizing fasciitis, and deep cervical infections, including retropharyngeal abscess, lymphadenitis, orbital cellulitis, endocarditis, and sepsis. Necrotizing pneumonia associated with CA-MRSA usually affects young, healthy patients, with high morbidity and mortality (8% - 100%).^(1,8,11,12)^ In a study that evaluated CA-MRSA necrotizing pneumonia cases, severe cases were associated with influenza-like illness 33% to 71% of the time. In the same study, PVL genes were found in 85% - 100% of these cases.^(1)^ They usually present with high fever and early-onset hemoptysis, rapidly progressing to ventilatory failure and septic shock, as our patient did. Leukopenia, also present in this case, is a frequent finding and a predictor of poor prognosis.^(1,11,12)^

Regarding treatment, in cases in which CA-MRSA-associated pneumonia is suspected, according to the local epidemiology and seasonality of influenza, early initiation of antimicrobial coverage is indicated, often in combination therapy, in addition to supportive therapy, adjusting the spectrum according to the results of subsequent cultures. In fulminant pneumonia caused by PVL-producing CA-MRSA, the use of a toxin inhibitor such as clindamycin, rifampicin, or linezolid is recommended. Combinations of vancomycin with clindamycin or rifampicin or of rifampicin with linezolid or clindamycin have been successful. Extracorporeal membrane oxygenation therapy can often be considered early in these cases.^(11)^

This case report is relevant because it highlights the presence of CA-MRSA in Brazil, as there are few studies on the prevalence of the disease in Brazil. It also adds to the understanding of the anatomopathological characteristics of CA-MRSA. A limitation of this study was the impossibility of evaluating the bacteriological characteristics of CA-MRSA, including the strain and the presence of virulence factors, such as PVL. However, the clinical course and the pathological findings are compatible with the PVL-producing variant of CA-MRSA. For conclusive diagnoses, it is necessary to investigate the specific genes *lukS-PV* and *lukF-PV* by polymerase chain reaction, which are not available at the hospital where the patient was treated.

This case is noteworthy for its fulminant and dramatic infection in a previously healthy young patient. Unfortunately, upon arrival at the tertiary hospital for ICU care, the patient already had signs of end-stage septic and hypovolemic shock, as recorded in the anatomopathological examination. It is important to be aware of the risk factors, epidemiological conditions, and clinical presentation of CA-MRSA to start appropriate antimicrobial treatment as early as possible, in addition to supportive therapy.
